# Tocilizumab improves survival in severe COVID-19 pneumonia with persistent hypoxia: a retrospective cohort study with follow-up from Mumbai, India

**DOI:** 10.1186/s12879-021-05912-3

**Published:** 2021-03-05

**Authors:** Yojana Gokhale, Rakshita Mehta, Uday Kulkarni, Nitin Karnik, Sushant Gokhale, Uma Sundar, Swati Chavan, Akshay Kor, Sonal Thakur, Trupti Trivedi, Naveen Kumar, Sujata Baveja, Aniket Wadal, Shaonak Kolte, Aukshan Deolankar, Sangeeta Pednekar, Lalana Kalekar, Rupal Padiyar, Charulata Londhe, Pramod Darole, Sujata Pol, Seema Bansode Gokhe, Namita Padwal, Dharmendra Pandey, Dhirendra Yadav, Anagha Joshi, Harshal Badgujar, Mayuri Trivedi, Priyanshu Shah, Prerna Bhavsar

**Affiliations:** 1 Lokmanya Tilak Municipal Medical College, Sion Mumbai, 400022 India; 2 Christian Medical College, Vellore, Tamil Nadu India

**Keywords:** IL-6, Interlukin-6, Cytokine storm, Hyperinflammatory syndrome, CO-RADS, CT-severity score

## Abstract

**Background:**

Cytokine storm triggered by Severe Coronavirus Disease 2019 (COVID-19) is associated with high mortality. With high Interleukin -6 (IL-6) levels reported in COVID-19 related deaths in China, IL-6 is considered to be the key player in COVID-19 cytokine storm. Tocilizumab, a monoclonal antibody against IL-6 receptor, is used on compassionate grounds for treatment of COVID-19 cytokine storm. The aim of this study was to assess effect of tocilizumab on mortality due to COVID-19 cytokine storm.

**Method:**

This retrospective, observational study included patients of severe COVID-19 pneumonia with persistent hypoxia (defined as saturation 94% or less on supplemental Oxygen of 15 L per minute through non-rebreathing mask or PaO2/FiO2 ratio of less than 200) who were admitted to a tertiary care center in Mumbai, India, between 31st March to 5th July 2020. In addition to standard care, single Inj. Tocilizumab 400 mg was given intravenously to 151 consecutive COVID-19 patients with persistent hypoxia, from 13th May to 5th July 2020. These 151 patients were retrospectively analysed and compared with historic controls, ie consecutive COVID-19 patients with persistent hypoxia, defined as stated above (*N* = 118, from our first COVID-19 admission on 31st March to 12th May 2020 i.e., till tocilizumab was available in hospital). Univariate and multivariate Cox regression analysis was performed for identifying predictors of survival. Statistical analysis was performed using IBM SPSS version 26.

**Results:**

Out of 269 (151 in tocilizumab group and 118 historic controls) patients studied from 31st March to 5th July 2020, median survival in the tocilizumab group was significantly longer than in the control group; 18 days (95% CI, 11.3 to 24.7) versus 9 days (95% CI, 5.7 to 12.3); log rank *p* 0.007. On multivariate Cox regression analysis, independent predictors of survival were use of tocilizumab (HR 0.621, 95% CI 0.427–0.903, *P* 0.013) and higher oxygen saturation.

**Conclusion:**

Tocilizumab may improve survival in severe COVID-19 pneumonia with persistent hypoxia. Randomised controlled trials on use of tocilizumab as rescue therapy in patients of severe COVID-19 pneumonia with hypoxia (PaO2/FiO2 less than 200) due to hyperinflammatory state, are warranted.

## Background

In December 2019, a newly discovered coronavirus, SARS-CoV-2 caused the novel coronavirus disease (COVID-19), that spread rapidly to become a pandemic. Around 80% patients have a mild course and overall case fatality rate is 2–3%, around 20% need hospitalization (14% have severe disease and 5% are critical) [1]. High IL-6 levels are reported in COVID-19 related deaths in China [2]. Therefore IL-6 is considered to be the key player in COVID-19 cytokine storm an entity characterized by fever, hypoxia (due to acute lung injury with lung infiltrates on imaging and raised inflammatory markers). Mortality is high in those with severe acute lung injury. With absence of specific antiviral drugs, treatment is essentially empirical, supportive and symptomatic, with

- antiviral - Lopinavir/ Ritonavir [3], Remdesivir [4], Favipiravir, Oseltamavir, Interferons, Ivermectin [5] (in vitro reduction in viral load)
- Convalescent plasma with passive antiviral antibodies transfer,
- drugs to reduce virus induced inflammatory response including Methyl Prednisolone and IL-6 blockade with Tocilizumab [6–9], JAK-inibitors [10]
- conventional or Low Molecular Weight Heparin (LMWH) for virus induced coagulopathy [11, 12]
- Hydroxychloroquin and Azithromycin
- supportive – Oxygen therapy/ high flow nasal cannula, non-invasive ventilation, mechanical ventilator, extracorporeal membrane oxygenation, antibiotics (to treat secondary infection), inotropic support and renal replacement.

Autopsy studies from deaths due to coronavirus infection suggested that aberrant host immune response results in a lethal inflammatory cytokine storm [13]. Increased alveolar exudates caused by aberrant host immune response and inflammatory cytokine storm probably impedes alveolar gas exchange and contributes to the mortality of severe COVID-19 patients. IL-6 is one of the most important cytokines involved in COVID-19 cytokine storm. Therefore tocilizumab, a humanized monoclonal antibody against IL-6 receptor (IL-6R) is investigated in treatment of seriously ill COVID-19 patients with cytokine storm. Untreated cytokine storm can progress from respiratory failure, to cardiovascular collapse, multiorgan dysfunction, and death.

## Methods

Study design and participants: This is a retrospective, observational study done at a single tertiary care center in Mumbai, India. The study population included adults of age more than 18 years with a positive nasopharyngeal swab for COVID-19 by RT-PCR, admitted from 31st March-5th July 2020.

Inclusion criteria were:

- persistent hypoxia (defined as saturation 94% or less on supplemental Oxygen of 15 L per minute through non-rebreathing mask or PaO2/FiO2 ratio of less than 200),
- bilateral pulmonary infiltrates on chest x-ray and
- raised inflammatory markers (C-Reactive Protein, Lactose Dehydrogenase, Ferritin)

Tocilizumab was available to us (free through the Municipal Corporation of Greater Mumbai) from 13th May onwards and was used in patients satisfying inclusion criteria from 13th May to 5th July. Although all three inclusion criteria were mandatory in tocilizumab group, in the historic control group the only two mandatory inclusion criteria applied were persistent hypoxia as defined above and presence of bilateral alveolar shadows. High C-reactive Protein could not be applied as inclusion criterion in the historic control group due to non availability of this test in our institute at that time, with prevalent financial constraints at that time.

Exclusion Criteria were:

- altered sensorium
- hypotension requiring multiple inotropic agents or multisystem organ failure (MSOF)
- patients suffering from terminal malignancy
- cardiomyopathy with ejection fraction less than 20%

Clinical features, co-morbidities, laboratory investigations and treatment details of all patients satisfying inclusion criteria were recorded. We have follow up of 55 days after last patient enrolment. Historic control group consisted of patients with COVID-19 infection satisfying inclusion criteria, from 31st March to 12th May (i.e., from first COVID-19 admission to our hospital till tocilizumab became available). Their data was obtained from indoor papers medical records. The study was approved by the Institutional Ethical Committee. Written consent for compassionate use of tocilizumab was obtained from patient or relative, and a consent waiver was permitted by institutional ethics committee for this retrospective study.

### Procedures

All patients received standard treatment consisting of antibiotics (Piperacilin-Tzobactum or Meropenem/ Vancomycin in view of critical condition), hydroxychloroquine 400 mg once daily, ivermectin 12 mg once daily, oseltamivir 75 mg twice daily, low molecular weight heparin 1 mg/ kg subcutaneously once daily (twice daily if D-dimer > 3000 ng/mL), methylprednisolone 125–500 mg intravenously once daily, and other supportive care as needed (Oxygen through non-rebreathing mask, High Flow Nasal Canula, Noninvasive or invasive ventilator support, inotropic support, renal replacement therapy). In addition to standard treatment, tocilizumab group received single intravenous dose of 400 mg tocilizumab.

### Outcomes: primary outcome was death or recovery

Statistical analysis: Comparison of the characteristics of the patients who received tocilizumab versus the control group, and comparison of characteristics of patients who survived versus those who died was performed. For comparison of categorical data, chi square test was used while for ordinal or continuous data, independent samples Mann Whitney U test was used. A *p* value of less than 0.05 was considered as significant.

Univariate and multivariate Cox regression analysis and logistic regression were performed for identifying predictors of survival. Log rank test was used to compare survival between patients who received tocilizumab versus the control group. Survival time was calculated from the date of giving tocilizumab to avoid immortal time bias. Statistical analysis was performed using IBM SPSS version 26.

## Results

From 31st March to 5th July 2020, a total of 2183 COVID-19 patients were admitted under Medicine department. Three hundred and ninety seven had persistent hypoxia (defined as saturation 94% or less on supplemental Oxygen of 15 L per minute through non-rebreathing mask or PaO2/FiO2 ratio of less than 200); of them 128 died within 24 h of admission and were not included in the study. A total of 269 patients with persistent hypoxia were included in the study. One fifty one received single intravenous infusion of tocilizumab 400 mg and 118 who did not, were historic controls.

Their characteristics are shown in Table 1. Tocilizumab group was younger (53 years v/s 55 years), but had lower mean Oxygen saturation of 86% (82–92%) v/s 91% (88–93%) in the control group. In tocilizumab group 63.6% had at least one co-morbidity and 36.4% were without any co-morbidity, whereas in the control group 74.6% had at least one co-morbidity and 25.4% were without any comorbidity. Tocilizumab group had more patients with obesity and less proportion of patients with hypertension than the control group.

**Table 1:** Comparison of the characteristics of the patients who received tocilizumab versus those who did not

Variable	Tocilizumab (*n* = 151)	Control (*n* = 118)	*P* value
Age	53 (44–60)	55 (47–64)	0.007
Male sex	107 (70.9)	69 (58.5)	0.034
Hypertension	40 (26.5)	53 (44.9)	0.002
Diabetes	78 (51.7)	59 (50)	0.788
Obesity	14 (9.3)	0 (0)	0.001
Other comorbidities	5 (3.3)	13 (11)	0.012
Number of comorbidities	1 (0–1)	1 (1–2)	0.031
No comorbidities	55 (36.4)	28 (23.7)	0.035
Category^a^			0.036
E	125 (82.8)	108 (91.5)	
F	26 (17.2)	10 (8.5)	
Oxygen saturation	86 (82–92)	91 (88–93)	0.001
Respiratory rate	34 (30–40)	30 ((27–38)	0.137
Serum creatinine	1 (1–1.9)	1.3 (1–1.7)	0.002
Non-invasive ventilation	56 (37.1)	0 (0)	0.001
Invasive ventilation	22 (14.6)	8 (6.8)	0.044
Deaths	79 (52.3)	74 (62.7)	0.088

^a^Revised guidelines on clinical management of COVID-19.Ministry of health & family welfare,directorate general of health services, government of India, (2020). https://www.mohfw.gov.in/pdf/Revised National Clinical Management Guideline for-COVID1931032020.pdf

**Fig. 1:**
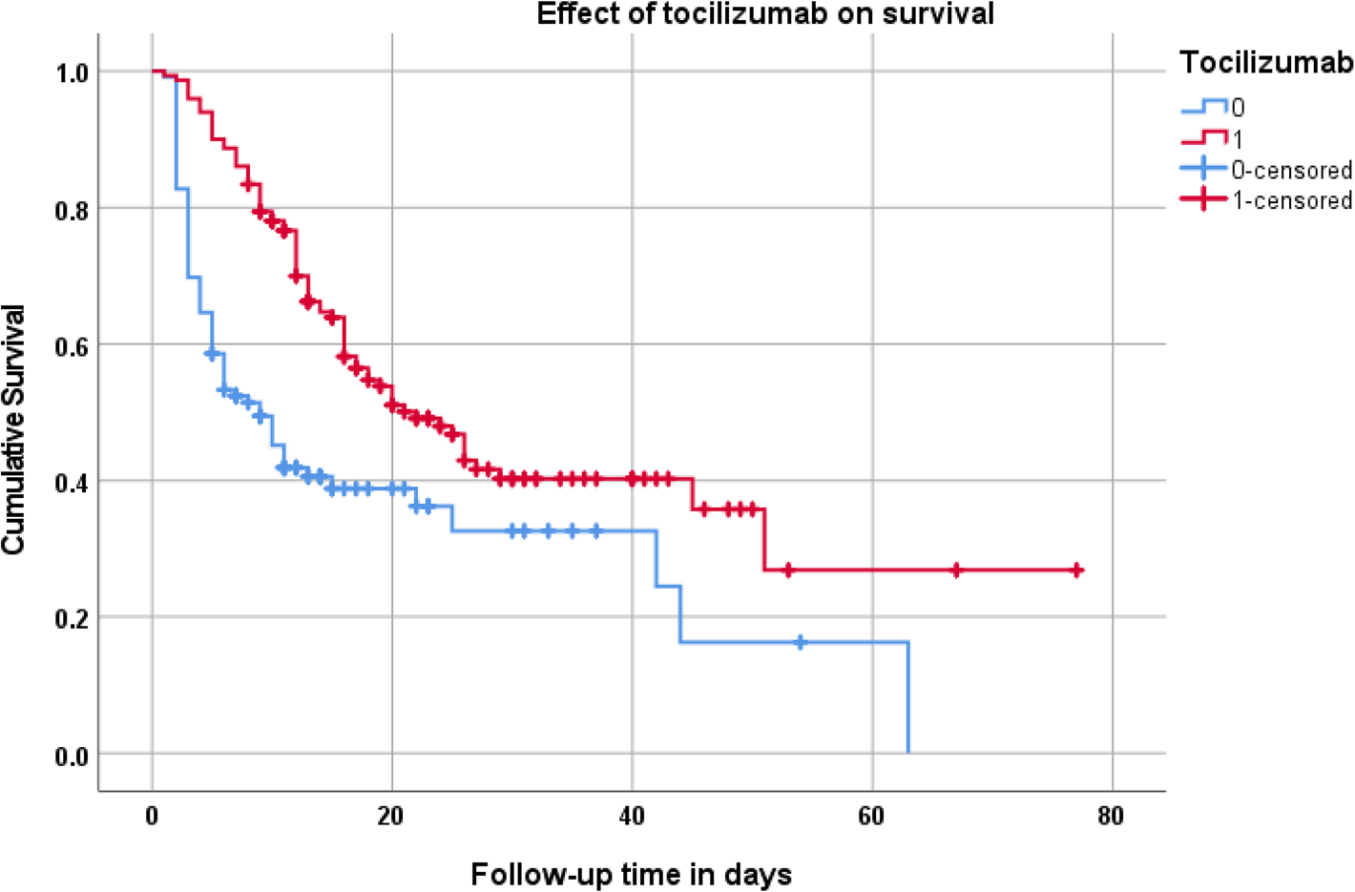
Survival analysis show the effect of tocilizumab on survival. (The median survival in the tocilizumab group was significantly longer than in the control group; 18 days (95% CI: 11.3 to 24.7) versus 9 days (95% CI: 5.7 to 12.3); log rank *p* 0.007)

Non invasive ventilation was used in 56/151 patients from tocilizumab group (15 of them later required invasive ventilation) but in none from control group (as a rule, it was avoided initially due to fear of aerosolization with increased risk to health care workers). Overall, 30 patients required invasive ventilation (22 from tocilizumab group and 8 from control group). Inotropic support was required in 11 patients from tocilizumab group and 7 patients from control group. In tocilizumab group, 79 out of 151 died (52.3% mortality) and in control group 74 out of 118 died (62.7%). Figure 1 depicts effect of tocilizumab on overall survival. (The median survival in the tocilizumab group was significantly longer than in the control group; 18 days (95% CI: 11.3 to 24.7) versus 9 days (95% CI: 5.7 to 12.3); log rank *p* 0.007). From tocilizumab group 72 out of 151 patients (47.7%) were discharged, whereas from the control group 44 out of 118 (37.3%) were discharged. Tocilizumab was well tolerated and no adverse drug reactions were noted.

Table 2 shows comparison of the demographic and laboratory parameters in ‘overall’ survived versus non-survived groups (including both control and tocilizumab groups). Those who survived were significantly younger (52 v/s 55 years, *p* = 0.029) and had significantly higher Oxygen saturation (91% v/s 88%, *p* = 0.002), lower respiratory rate (30 v/s 36 breaths per min, *p* = 0.001) and lower serum creatinine (1 mg/dl v/s 1.3 mg/dl, *p* = 0.001). The higher average serum creatinine in the non-survived group probably reflected some degree of hypotension with prerenal element.

**Table 2:** Comparison of the characteristics of the patients who survived versus those who did not (both tocilizumab and control groups)

Variable	Survived (*n* = 116)	Died (*n* = 153)	*P* value
Age	52 (44–60)	55 (47–62)	0.029
Male sex	71 (61.2)	105 (68.6)	0.205
Hypertension	37 (31.9)	56 (36.6)	0.422
Diabetes	55 (47.4)	82 (53.6)	0.315
Obesity	3 (2.6)	11 (7.2)	0.092
Other comorbidities	8 (6.9)	10 (6.5)	0.907
Number of comorbidities	1 (0–1)	1 (0–2)	0.074
Category			0.10
E	105 (90.5)	128 (83.7)	
F	11 (9.5)	25 (16.3)	
Oxygen saturation	91 (86–93)	88 (83–92)	0.002
CRP	97.5 (63.5–159)	90 (56–136)	0.264
Respiratory rate	30 (30–36)	36 (30–40)	0.001
Serum Creatinine	1 (1–1.2)	1.3 (1–2)	0.001
SGOT	49 (37–75)	57 (42–72)	0.169
SGPT	40 (30–58)	44 (28–68)	0.977
LDH	666 (275–990)	978 (369–2000)	1.000
Ferritin	437 (293–947)	690 (369–1257)	0.364
D-dimer	1000 (1000–1927)	1411 (1000–5000)	0.079
IL-6	455 (75–984)	Not available	
WBC counts (× 10^9^/L)	8.9 (5.85–13.6)	8.55 (6.1–12.15)	0.799
Platelet count (×10^9^/L)	200 (200–300)	200 (200–300)	0.314
CT CORAD	6 (6–6)	6 (6–6)	1.000
CT severity score	21 (17–24)	24 (21–25)	0.343
Thrombosis on CT	2 of 8	1 of 2	0.490
Tocilizumab	72 ((62.1)	79 (51.6)	0.088
Day of tocilizumab	3 (2–6)	3 (2–5)	0.865
Non-invasive ventilation	22 (19)	34 (22.2)	0.515
Invasive ventilation	1 (0.9)	29 (19)	0.001

Our patients, on the whole, did not have significant leucopenia (white blood cells less than 4000), lymphopenia or thrombocytopenia. Inflammatory markers like CRP (mean 93.75, range 56–159, normal < 6 mg/L), LDH (mean 822, range 275–2000 U/L) and serum ferritin (mean 563.5, range 293–1257 ng/ml) were markedly elevated in both groups and were not statistically different in those who survived and those who didn’t.

D-dimer level was higher in the ‘not-survived’ group, (mean 1411 ng / ml, range of 1000–5000 ng/ ml) than in those who survived (mean 1000 ng / ml, range of 1000–1927 ng / ml), but the difference was not statistically significant (*p* 0.079).

Total 38 radiological scans (High Resolution CT chest: 26, CT-Pulmonary Angiography: 9, CT-brain: 3) were done in 28 out of 151 patients receiving Tocilizumab. Of these 28 patients, 21 were not on any form of advanced respiratory support at any time. Seven patients had radiological scans done early in the disease and ultimately required advanced respiratory support (HFNC/ NIV/ ventilator). Only 7 out of 68 patients who were on advanced respiratory support (HFNC/ NIV/ Ventilator) had radiological scans done before getting switched to the same. In the 28 patients with radio-imaging available 11 patients expired and 17 were discharged. The CT severity scores were similar in the two groups (median of 21 versus 24; *p* value of 0.343). The median CORAD score was also 6 in both groups (*p* 1.000).

Table 3 depicts univariate and multivariate Cox regression analysis. Data on CRP, SGOT, SGPT, LDH, IL-6, WBC and differential count, platelet count, ferritin and d-dimer was not available in all patients in the control group. Data on respiratory rate was available only for 142 patients and d-dimer data was available for 78 patients. Hence these parameters were not included in the multivariate analysis. On multivariate analysis, ‘older age’ was not detected to be a risk factor for death. Survival was not different in those with or without any co-morbidity.

**Table 3:** Univariate and multivariate cox regression analysis

Variable	Univariate			Multivariate		
	HR	95%CI	*P* value	HR	95%CI	*P* value
Age	1.017	1.002–1.032	0.029	1.012	0.994–1.029	0.200
Male Sex	0.813	0.577–1.145	0.237			
Hypertension	0.887	0.638–1.233	0.476			
Diabetes	0.983	0.714–1.353	0.916			
Obesity	0.805	0.435–1.489	0.489			
Other comorbidities	0.926	0.487–1.761	0.815			
Number of comorbidities	1.090	0.903–1.315	0.371			
Oxygen saturation	0.979	0.962–0.996	0.017	0.969	0.950–0.989	0.002
CRP	0.999	0.996–1.002	0.379			
Creatinine	1.157	1.031–1.298	0.013	1.123	0.995–1.267	0.061
Respiratory rate	1.045	1.006–1.084	0.023			
LDH	1.000	0.997–1.003	0.880			
Ferritin	1.000	1.000–1.001	0.626			
D-dimer	1.000	1.000–1.000	0.043			
WBC counts	1.000	1.000–1.000	0.875			
Tocilizumab	0.655	0.476–0.901	0.009	0.621	0.427–0.903	0.013
CT severity score	1.084	0.810–1.449	0.588			
Thrombosis	2.160	0.135–34.608	0.586			
Non-invasive ventilation	0.770	0.525–1.131	0.183			
Invasive ventilation	2.028	1.349–3.049	0.001	2.31	1.442–3.701	0.001

Data on respiratory rate was available only for 142 patients while d-dimer was available for 78 patients. Hence these parameters were not included in the multivariate analysis

The independent predictors of survival were use of tocilizumab (HR 0.621, 95% CI 0.427–0.903, *P* 0.013), higher oxygen saturation (HR 0.969, 95% CI 0.950–0.989, *p* 0.002) and use of invasive ventilation (HR 2.31, 95% CI: 1.442–3.701, *p* 0.001) on multivariate analysis.

Table 4 depicts comparison of the characteristics of the patients who survived (*N* = 72) versus those who did not (*N* = 79), in the tocilizumab group. Tocilizumab was administered on 2nd to 5th day of admission (average 3rd day) in both groups. Those who ‘survived’ had higher Oxygen saturation than ‘non-survived group’ (mean 88% with a range of 85–93% v/s mean 85% and range of 79–90%- *p* = 0.014) and were less tachypnic than ‘non-survived group’(respiratory rate 30 v/s 36 breaths per min, *p* = 0.002),at the time of enrolment for tocilizumab. Obesity and raised serum creatinine, on the other hand, had adverse effect on survival, *p* = 0.039 and 0.001 respectively. Blood levels of inflammatory markers were comparable in both groups. D-dimer was higher in those who did not survive than in those who survived, but the difference was not statistically significant. Proportion of patients who required invasive ventilation was significantly more amongst patients who died as compared to those who survived (26.6% versus 1.4%, *p* 0.001).

**Table 4:** Comparison of the characteristics of the patients receiving tocilizumab who survived versus those who did not

Variable	Survived (*n* = 72)	Died (*n* = 79)	*P* value
Age	52 (42–59)	55 (46–60)	0.105
Male sex	48 (66.7)	59 (74.7)	0.279
Hypertension	19 (26.4)	21 (26.6)	0.979
Diabetes	33 (45.8)	45 (57)	0.172
Obesity	3 (4.2)	11 (13.9)	0.039
Other comorbidities	2 (2.8)	3 (3.8)	0.727
Number of comorbidities	1 (0–1)	1 (0–2)	0.082
Category			0.143
E	63 (87.5)	62 (78.5)	
F	9 (12.5)	17 (21.5)	
Oxygen saturation	88 (85–93)	85 (79–90)	0.014
CRP	97.5 (63.5–159)	90 (56–136)	0.264
Respiratory rate	30 (30–36)	36 (30–40)	0.002
Serum Creatinine	1 (1–1)	1 (1–2)	0.001
SGOT	49 (37–75)	57 (42–72)	0.169
SGPT	40 (30–58)	44 (28–68)	0.977
LDH	701 (515–988)	608 (462–753)	1.000
Ferritin	437 (293–947)	978 (369–2000)	0.364
D-dimer	1000 (1000–1927)	1411 (1000–5000)	0.079
IL-6	455 (75–984)	Not available	
WBC counts (×10^9^/L)	8.9 (5.85–13.6)	8.4 (6.1–12.0)	0.799
Neutrophil percentage	72 (65–78)	72 (70–75)	1.000
Lymphocyte percentage	25 (17–29)	23 (10–27)	0.469
Platelet count (×10^9^/L)	200 (200–300)	200 (200–300)	0.314
Day of tocilizumab	3 (2–6)	3 (2–5)	0.865
Non-invasive ventilation	22 (30.6)	34 (43)	0.113
Invasive ventilation	1 (1.4)	21 (26.6)	0.001

## Discussion

We found a significant reduction in risk of death in severe COVID-19 pneumonia with persistent hypoxia receiving a single dose of intravenous tocilizumab 400 mg, compared with those treated with standard care alone. The hazards of dying in the tocilizumab group were 0.621 times of that in the control group. We reported 47.1% mortality in our first 70 patients of severe COVID-19 pneumonia with persistent hypoxia treated with tocilizumab till 5th June 2020, compared with 67% mortality in 90 controls (3 weeks prior to availability of tocilizumab) with persistent hypoxia due to severe COVID pneumonia [14]. At 3 weeks follow-up 11 / 151(15.7%) patients were still hospitalized. Two of them died later, increasing mortality to 50% in tocilizumab group. In a retrospective observational study in COVID 19 patients treated in ICU in New Jersey, Noa et al. [15] reported 49% mortality in 210 patients treated with tocilizumab and 61% mortality in 420 patients who did not receive tocilizumab. In present study, 60 out of our 151 patients from tocilizumab group were managed in COVID ICU. Due to non-availability of COVID ICU beds, 15 patients received non-invasive ventilation (NIV) in covid wards, and 7 patients received oxygen through high flow nasal canula (HFNC) in covid wards. Non-invasive ventilation was used in 56 patients from tocilizumab group (15 of them later required invasive ventilation) but in none from control group (it was avoided initially due to fear of aerosolization causing increased risk to health care workers). Overall, 30 patients required invasive ventilation (22 from tocilizumab group and 8 from control group. Many investigators, for example Kewan [9], Nicola [8] have used early Tocilizumab in Covid 19 treatment. Nicola et al. [8] used tocilizumab in patients with peripheral Oxygen saturation 93% on room air or PaO2/FiO2 less than 300 mm of Hg, and documented reduction in mortality from 50 to 7.7%. With early use of tocilizumab, Nicola et al. [8] could reduce risk of death by 94%. Although more than 70% of our admitted patients were hypoxic (Oxygen saturation less than 95% on ambient air) during hospital stay, the limited availability of tocilizumab made it mandatory for us to formulate strict inclusion criteria for tocilizumab administration. These criteria were formulated by consensus of department members and approved by institutional ethics committee. (saturation 94% or less on 15 L per min supplemental Oxygen through non-rebreathing mask or PaO2/FiO2 less than 200). Depending upon inclusion criteria for use of tocilizumab in severe Covid19 pneumonia, severity of disease at the time of intervention and primary outcomes various outcomes are reported. Guaraldi et al. [7] reported mortality in tocilizumab versus standard care group to be 7 and 20% respectively (*P* < .0001), with inclusion criteria being respiratory rate more than or equal to 30 per min, Oxygen saturation less than or equal to 93% on room air or PaO2/FiO2 ratio being 300 or less, and bilateral lung infiltrates more than 50% being present within 24 to 48 of admission. Average PaO2/FiO2 ratio in their study was 169 in tocilizumab group as against 277 in standard care group. They also reported the effect of tocilizumab was at least two times higher in people with a baseline PaO2/FiO2 ratio of less than 150 mmHg, implying that the benefit of tocilizumab could be greater in patients with a greater risk of death or mechanical ventilation [7]. Rossotti et al. [16] reported tocilizumab use to be associated with a better overall survival (HR 0.499 [95% CI 0.262–0.952], *p* = 0.035) as compared to control, their inclusion criteria being respiratory rate more than or equal to 30 per min, Oxygen saturation less than or equal to 93% on room air or PaO2/FiO2 ratio being 300 or less. In STOP-COVID trial [17] (large multicenter observational comparative study from USA, in ICU admitted Covid-19 patients receiving tolicizumab within 48 h of ICU admission versus non-tocilizumab cohort, with 419 and 3492 patients respectively after inverse probability weighting to match baseline characteristics and severity of illness, retrospectively analyzed), Gupta et al., reported 28.9% mortality in those treated with tocilizumab and 40.6% in not treated with tocilizumab. The risk of death was lower in the group of patients treated with tocilizumab as compared to those not treated with the same (HR, 0.71; 95%CI, 0.56–0.92.).On further stratification as per severity of hypoxemia, i.e. PaO2/FiO2 > 200 on ICU admission or patients not on mechanical ventilators had HR 0.88 [95% CI, 0.58–1.35] as against that for patients with PaO2/FiO2 < 200 or on mechanical ventilator had HR 0.59 [95% CI, 0.43–0.81]. The recently published Randomized Evaluation of COVID-19 Therapy (RECOVERY) trial [18] found that dexamethasone reduces mortality in hospitalized patients with COVID-19. The beneficial effect of dexamethasone was particularly pronounced in patients receiving invasive mechanical ventilation. These early data suggest that medications targeting dysregulated inflammation may be a promising therapeutic strategy among critically ill patients with COVID-19. But in published randomized control trials on tocilizumab in Covid19, the effect of tocilizumab on mortality are discordant with the results of observational studies. Possible reasons could be study design, different study population, severity of illness and timing of administration of tocilizumab.

So far 5 RCTs are completed [19–23]. In BACC (randomized, double-blind, placebo-controlled) trial [19], 16% patients did not require supplemental O2, 80% required less than 6 L per min O2. Though average saturation and PaO2/FiO2 ratio is not mentioned, over 90% patients had mild disease severity. The hazard ratio for intubation or death in the tocilizumab group as compared with the placebo group was 0.83 (95% confidence interval [CI], 0.38 to 1.81; *P* = 0.64). Authors have not ruled out possibility of some benefit or harm due to wide CI, though tocilizumab was not effective for preventing intubation or death. In the sample size calculations for this trial, the authors assumed an event rate for the primary outcome of 30% in the placebo group and 15% in the tocilizumab group. However, only 27 patients (11.2%) had a primary-outcome event (19 [7.8%] were intubated and 8 [3.3%] died without having been intubated. The event rate was lower than anticipated, which probably affected the interpretation of effect of treatment [24].. In CORIMUNO-TOCI-19 (open label RCT) by Hermine et al. [20], patients with PaO2/FiO2 200-300 were included, whereas those from ICU/ on HFNC/NIV/MV (mechanical ventilator) were excluded, on day 14 12% reduction in need for MV or death was reported, but no reduction in death on day 28. In RCT-TCZ-COVID-19 by Salvarani et al. [21] included patients with PaO2/FiO2 200-300, average being 264, with average CRP 8.2(much less elevation as compared to our cohort with average CRP of 93.75), there was no benefit in achieving primary outcome (death/ MV/ PaO2:FiO2 < 150) with tocilizumab. In this study among the 17 patients reaching one primary endpoint (PaO2/FiO2 < 150) in the standard care group, 14 received tocilizumab as a rescue therapy (as per protocol). At 30 days, the incidence of intubation and death was comparable between the 2 groups. One can infer that the early administration of tocilizumab does not provide any significant advantage in reduction of intubation or mortality over a deferred administration at PaO2/FiO2 ratio less than 150 mmHg. In COVACTA trial [22] (randomized, double-blind, placebo-controlled), which reported no mortality benefits on 28 day with tocilizumab, eligibility criteria were broad, viz. patients with saturation less than 94% on room air, which also enrolled 37% patients on MV.

Regarding EMPACTA [23], it included patients with saturation less than 94% at room air. EMPACTA trial, with similar inclusion criteria was conducted in minority groups (blacks, Hispanics, Asians), who have higher risk of death, 44% reduction in need for mechanical ventilator or death was reported.

Two RCTs viz. BACC (USA) [19] and RCT-TCZ-COVID-19 (Italy) [21] reported 3.3 and 2.4% mortality respectively. Whereas reported mortality for hospitalized Covid-19 patients in USA [25] and Italy [26] is 22.6 and 29.7% respectively, implying thereby that these RCTs enrolled mild cases of Covid-19 for tocilizumab usage.

Tleyhej et al. [27] performed a meta-analysis of 5 RCTs (1325 patients) and 18 cohort studies (9850 patients). They noted that the RCTs did not show reduction of short term mortality with the use of tocilizumab. However cohort studies showed an association of tocilizumab use with reduced mortality. They also reported a cumulative moderate-certainty evidence of reduction in the risk of mechanical ventilation in hospitalized COVID-19 patients with the use of tocilizumab. For detecting a RR of 0.73 for mortality with 80% power and 5% significance, the sample size required for an RCT is 4506, while the total number of patients in the five RCTs is 1325.

Press release from ongoing REMAP-CAP trial [28] on 19th November stated tocilizumab was 99% more likely to reduce deaths and time spent in intensive care among critically ill patients with severe COVID-19, compared to patients who did not receive the treatment. REMAP-CAP trial inclusion criteria are patients admitted to ICU with severe pneumonia requiring respiratory support such as high-flow nasal oxygen, continuous positive airway pressure (CPAP) or non-invasive ventilation, or invasive mechanical ventilation and COVID-19 infection confirmed by microbiological testing or where a multidisciplinary team has a high level of confidence that the clinical and radiological features suggest that COVID-19 is the most likely diagnosis). On 25th November, Interim position statement [29] given by NHS England is, ‘until the full data from the REMAP-CAP and RECOVERY trials are available, the off-label use of tocilizumab within critical care should follow the criteria and information described in this interim clinical position. The trial Data and Safety Monitoring Board (DSMB) has determined that it is ethically imperative to withdraw the standard-of-care control arm of the immune modulator domain of the REMAP-CAP trial.’

In the current study, clinical response in terms of reduction in Oxygen requirements and respiratory rate was observed within 24–72 h of tocilizumab infusion in those who responded. C-reactive Protein improved by day 3 to 4. Amongst patients who received tocilizumab, D-dimer levels were higher in ‘non-survived’ group than in ‘survived’ group. Although this difference was not statistically significant, suspicion of terminal pulmonary thromboembolic event was high on clinical grounds in the non-survived group. Zhou et al. [2] reported D-dimer more than 1000 nanogram/ml to be a risk factor for mortality. BACC supplementary data [19] has reported that coagulation abnormalities probably did not improve with tocilizumab. Our clinical impression is that patients in whom clinical improvement in terms of reduced Oxygen requirement did not occur had either extensive lung involvement or high D-dimer or pulmonary thrombi on CT-Pulmonary angiography (imaging could not be performed in all patients due to logistics issues in patients on High Flow Nasal Canula or on non-invasive or mechanical ventilators). In 26 patients, HRCT chest could be performed, all had CO-RAD score [30] 6 (1 lowest to 6 highest suspicion) and average CT-severity score [31] was 21 out of 25. A study from Wuhan [32] reported maximum CT score higher than 11 was associated with development of severe illness. CT-pulmonary angiography was performed in 9 patients and documented pulmonary thrombi in 3 patients. CT-brain was performed in 3 patients and documented brain infracts in all. One patient with pulmonary thrombus developed two large infarcts in brain despite full dose heparin and streptokinase for pulmonary thrombus. Radiological imaging was not possible in more severely affected patients due to them being on advanced respiratory support.

The possibility of secondary infection due to immunosuppressants (steroids and tocilizumab), contributing to morbidity, also has to be considered in both Tocilizumab and control groups, though higher antibiotics like Piperacilin Tazobactum or Meropenem / Vancomycin were used in all critically ill patients. Procalcitonin levels could not be done due to cost constraints. Presence or absence of any co-morbidity did not affect primary outcome in the current study.

## Conclusion

Use of tocilizumab confers a significant survival benefit in COVID19 patients with persistent hypoxia despite optimal supportive care.

One of the limitations of this study was that tocilizumab group was overall younger, but this factor was likely to have been offset by lower average Oxygen saturation level in tocilizumab group, though propensity matching was not done.

These preliminary results are encouraging. Randomised controlled trials on use of tocilizumab as rescue therapy in patients of severe COVID-19 pneumonia with hypoxia (PaO2/FiO2 less than 200) due to hyperinflammatory state, are warranted.

## Data Availability

Raw data available upon reasonable request from the corresponding author.

## Abbreviations

COVID 19: Coronavirus Disease 2019
IL-6: Interleukin 6
NIV: Non-invasive ventilation
HFNC: High Flow nasal canula
MV: Mechanical ventilator
JAK: Janus kinase
RT-PCR: Real time polymerase chain reaction
CRP: C- reactive protein
SGOT: Serum glutamic-oxaloacetic transaminase.
SGPT: Serum glutamic-pyruvic transaminase
LDH: Lactose dehydrogenase
WBC: White blood Corpuscle
CO-RADS: Coronavirus disease 2019 (COVID-19) Reporting and Data System
CTPA: Computerised tomography pulmonary angiography
HRCT: High resolution computerised tomography

## References

[1] Wu Z, McGoogan JM (2020). Characteristics of and important lessons from the coronavirus disease 2019 (COVID-19) outbreak in China: summary of a report of 72 314 cases from the Chinese Center for Disease Control and Prevention. JAMA..

[2] Zhou F, Yu T, Du R, Fan G, Liu Y, Liu Z (2020). Clinical course and risk factors for mortality of adult inpatients with COVID-19 in Wuhan, China: a retrospective cohort study. Lancet..

[3] Owa AB, Owa OT (2020). Lopinavir/ritonavir use in Covid-19 infection: is it completely non-beneficial?. J Microbiol Immunol Infect.

[4] Grein J, Ohmagari N, Shin D, Diaz G, Asperges E, Castagna A (2020). Compassionate use of Remdesivir for patients with severe Covid-19. N Engl J Med.

[5] Heidary F, Gharebaghi R (2020). Ivermectin: a systematic review from antiviral effects to COVID-19 complementary regimen. J Antibiot (Tokyo).

[6] Toniati P, Piva S, Cattalini M, Garrafa E, Regola F, Castelli F (2020). Tocilizumab for the treatment of severe COVID-19 pneumonia with hyperinflammatory syndrome and acute respiratory failure: a single center study of 100 patients in Brescia, Italy. Autoimmun Rev.

[7] Guaraldi G, Meschiari M, Cozzi-Lepri A, Milic J, Tonelli R, Menozzi M (2020). Tocilizumab in patients with severe COVID-19: a retrospective cohort study. Lancet Rheumatol.

[8] De Rossi N, Scarpazza C, Filippini C, Cordioli C, Rasia S, Mancinelli CR (2020). Early use of low dose tocilizumab in patients with COVID-19: a retrospective cohort study with a complete follow-up. EClinicalMedicine..

[9] Kewan T, Covut F, Al-Jaghbeer MJ, Rose L, Gopalakrishna KV, Akbik B (2020). Tocilizumab for treatment of patients with severe COVID-19: a retrospective cohort study. EClinicalMedicine..

[10] La Rosée F, Bremer HC, Gehrke I, Kehr A, Hochhaus A, Birndt S (2020). The Janus kinase 1/2 inhibitor ruxolitinib in COVID-19 with severe systemic hyperinflammation. Leukemia..

[11] Abou-Ismail MY, Diamond A, Kapoor S, Arafah Y, Nayak L (2020). The hypercoagulable state in COVID-19: incidence, pathophysiology, and management. Thromb Res.

[12] Zhang Y, Xiao M, Zhang S, Xia P, Cao W, Jiang W (2020). Coagulopathy and antiphospholipid antibodies in patients with Covid-19. N Engl J Med.

[13] Channappanavar R, Perlman S (2017). Pathogenic human coronavirus infections: causes and consequences of cytokine storm and immunopathology. Semin Immunopathol.

[14] Gokhale Y, Mehta R, Karnik N, Kulkarni U, Gokhale S (2020). Tocilizumab improves survival in patients with persistent hypoxia in severe COVID-19 pneumonia. EClinicalMedicine..

[15] Biran N, Ip A, Ahn J, Go RC, Wang S, Mathura S (2020). Tocilizumab among patients with COVID-19 in the intensive care unit: a multicentre observational study. Lancet Rheumatol..

[16] Rossotti R, Travi G, Ughi N, Corradin M, Baiguera C, Fumagalli R (2020). Safety and efficacy of anti-il6-receptor tocilizumab use in severe and critical patients affected by coronavirus disease 2019: a comparative analysis. J Inf Secur.

[17] Gupta S, Wang W, Hayek SS, Chan L, Mathews KS, Melamed ML (2021). Association between early treatment with tocilizumab and mortality among critically ill patients with COVID-19. JAMA Intern Med.

[18] RECOVERY Collaborative Group, Horby P, Lim WS, Emberson JR, Mafham M, Bell JL, Linsell L, et al. Dexamethasone in Hospitalized Patients with Covid-19 - Preliminary Report. N Engl J Med. 2020:NEJMoa2021436. 10.1056/NEJMoa2021436.

[19] Stone JH, Frigault MJ, Serling-Boyd NJ, Fernandes AD, Harvey L, Foulkes AS (2020). Efficacy of tocilizumab in patients hospitalized with Covid-19. N Engl J Med.

[20] Hermine O, Mariette X, Tharaux PL, Resche-Rigon M, Porcher R, Ravaud P, CORIMUNO-19 Collaborative Group (2021). Effect of tocilizumab vs usual care in adults hospitalized with COVID-19 and moderate or severe pneumonia: a randomized clinical trial. JAMA Intern Med.

[21] Salvarani C, Dolci G, Massari M, Merlo DF, Cavuto S, Savoldi L (2021). Effect of Tocilizumab vs standard care on clinical worsening in patients hospitalized with COVID-19 pneumonia: a randomized clinical trial. JAMA Intern Med.

[22] Rosas I, Bräu N, Waters M, Go RC, Hunter BD, Bhagani S, et al. Tocilizumab in hospitalized patients with COVID-19 pneumonia. medRxiv. 2020. 10.1101/2020.08.27.20183442.

[23] Salama C, Han J, Yau L, Reiss WG, Kramer B, Neidhart JD (2021). Tocilizumab in patients hospitalized with Covid-19 pneumonia. N Engl J Med.

[24] Huang E, Jordan SC (2020). Tocilizumab for Covid-19 - the ongoing search for effective therapies. N Engl J Med.

[25] Wang Z, Zheutlin A, Kao Y, Ayers K, Gross S, Kovatch P (2020). Hospitalised COVID-19 patients of the Mount Sinai Health System: a retrospective observational study using the electronic medical records. BMJ Open.

[26] Bellan M, Patti G, Hayden E, Azzolina D, Pirisi M, Acquaviva A (2020). Fatality rate and predictors of mortality in an Italian cohort of hospitalized COVID-19 patients. Sci Rep.

[27] Tleyjeh IM, Kashour Z, Damlaj M, Riaz M, Tlayjeh H, Altannir M, et al. Efficacy and safety of tocilizumab in COVID-19 patients: a living systematic review and meta-analysis. Clin Microbiol Infect. 2020: S1198-743X(20)30690-X. 10.1016/j.cmi.2020.10.036. 10.1016/j.cmi.2020.10.036 PMC7644182 33161150

[28] Tocilizumab effective in treating sickest patients of Covid19. Press Release from UMC UTRECHT & IMPERIAL COLLEGE LONDON, Nov 19, 2020. https://www.recover-europe.eu/tocilizumab-effective-in-treating-sickest-covid-19-patients/. Accessed 13 Dec 2020.

[29] Interim Position Statement for patients admitted to ICU with Covid-19 pneumonia (adults). https://www.cas.mhra.gov.uk/ViewandAcknowledgment/ViewAttachment.aspx?Attachment_id=103715. Accessed 13 Dec 2020.

[30] Prokop M, van Everdingen W, van Rees VT, Quarles van Ufford H, Stöger L, Beenen L (2020). CO-RADS: a categorical CT assessment scheme for patients suspected of having COVID-19-definition and evaluation. Radiology..

[31] Chang YC, Yu CJ, Chang SC, Galvin JR, Liu HM, Hsiao CH (2005). Pulmonary sequelae in convalescent patients after severe acute respiratory syndrome: evaluation with thin-section CT. Radiology..

[32] Xiao J, Li X, Xie Y, Huang Z, Ding Y, Zhao S (2020). Maximum chest CT score is associated with progression to severe illness in patients with COVID-19: a retrospective study from Wuhan, China. BMC Infect Dis.

